# Evaluation of bone availability for grafts in different donor sites, through computed tomography

**DOI:** 10.1590/1678-7757-2019-0435

**Published:** 2020-01-31

**Authors:** Géssyca Moreira Melo de Freitas GUIMARÃES, Gabriel Fiorelli BERNINI, Dayane Kemp GRANDIZOLI, Paulo Sergio Perri de CARVALHO, Eduardo Sanches GONÇALES, Osny FERREIRA

**Affiliations:** 1 Universidade de São Paulo Faculdade de Odontologia de Bauru Departamento de Cirurgia, Estomatologia, Patologia e Radiologia BauruSão Paulo Brasil Universidade de São Paulo, Faculdade de Odontologia de Bauru, Departamento de Cirurgia, Estomatologia, Patologia e Radiologia, Bauru, São Paulo, Brasil.

**Keywords:** Tomography, Surgery, Skull, Mandible

## Abstract

**Objective:**

To quantify the bone volume that can be safely withdrawn from 3 donor sites: (1) the mandibular symphysis, (2) the oblique mandibular line and (3) the skullcap.

**Methodology:**

For the symphysis, 200 tomographic exams were evaluated by the extension of the anterior loop of mental foramen, by the nerve, by the distance of the foramens, by the distance between the vestibular cortical and the lingual plates and by the distance between the apexes, or lower anterior teeth, and the mandibular base, using the “distance” tool of the I-CAT Vision, in the panoramic and parasagittal reformations. For the oblique line, 70 TCFC exams were analyzed retrospectively in panoramic and parasagittal reformations, evaluating the thickness of the vestibular cortical and the distance between the cortical and the mandibular canal. For the cranial bone, a hexagonal donor site located in parietal area was considered.

**Results:**

The average dimensions of the bone blocks that can be safely removed from the region of the mandibular symphysis are: 32.27 mm in length, 4.87 mm in height and 4 mm in thickness, providing a volume of 628.61 mm^3^ available for grafting. In the oblique line, the available bone volume for grafting was 859.61 mm^3^. In the region of the cranial vault, multiplying the average bone thickness by the area of the hexagon, an average volume of 2,499 mm^3^ was obtained.

**Conclusions:**

Comparing the donor sites, the bone availability in the cranial vault is 3 times greater than in the mandibular posterior region, and at least 2 times greater than in the mandibular symphysis.

## Introduction

The rehabilitation of edentulous patients has occupied a prominent place in Dentistry. Implantology offers excellent options for patients without enough bone to use a conventional prosthesis. However, for those with severe alveolar bone resorption, there is not enough bone for an implant installation. In these cases, bone grafts are required.^1^ For larger reconstructions, donor sites in extraoral bones are the most viable options due to the greater amount of bone available.

Autogenous bone grafts are often used to correct defects related to the bone volume of the recipient site, mainly because they are still considered the gold standard when compared with biomaterials. In individuals who have lost permanent teeth due to trauma, caries or periodontal diseases and who lack the required bone volume, the symphysis can provide an appropriate amount of bone for grafting, implant placement and prosthetic rehabilitation.^2^

Bone grafts are influenced by factors such as the surgical technique used, the bone quantity and quality of the donor site and the systemic conditions of the patients.^1^ The correct treatment planning, the adequate revision of the medical history, the absence of pathologies and deleterious habits, the proximity of the alveolar process to the location of anatomical structures – including maxillary sinus, nasal cavity, incisive canal (IC), mandibular canal (MC) and mental foramen (MF) –, and a well-executed surgical technique will reduce complications during the surgical procedure and increase its success rate.^3 , 4^

The region of the mandibular body and ramus, constituted by the cortical and trabecular bones, is one of the most used intraoral donor sites for this purpose, primarily for its bone quality. This provides osteogenesis, osteoconduction, osteoinduction and osteointegration, as well as low morbidity and few postoperative sensorial complaints when compared with other donor sites. Besides having a high concentration of bone morphogenetic proteins,^5^ this region has low volume loss and excellent incorporation in the short term. Another advantage is that the donor and the recipient sites are in the same surgical field, reducing the surgical time and the necessary amount of anesthetic and allowing the surgery to be performed at outpatient level. However, the access may reveal difficulties related to visibility and limitations on the graft size and shape,^6^ impairing the bone volume.^7^

Most studies on this subject^1 , 8 - 12^ report an advantage of the skullcap toward the other sites because it is a corticalized bone that undergoes less resorption, leading to more predictable results for the installation of implants, both in the maxilla and mandible, with lower postoperative morbidity. The disadvantages are related to the need for general anesthesia, to the potential complications and to the patient acceptance of cranial surgery more than to its surgical difficulty.^13^

As in any type of surgery, careful planning is essential; therefore, three-dimensional analysis using computed tomography is very useful.^1^ Cone-beam computed tomography (CBCT) is a diagnostic imaging method, especially indicated to examine the dentomaxillofacial complex,^4^ which enables the reformation of the maxillofacial bones without distortion and image-guided radiation dosing, with reduced costs.^4^ This examination technique improves the visualization of images and structures in a way that was not possible with the conventional radiography.^4^ Thus, this visualization capacity was used to quantify the bone availability, since studies that inform and discuss the bone volume that can be removed were not found in the scientific literature.

## Methodology and Results

### Mandibular symphysis

The sample size calculation was done according to some inclusion criteria. This study was approved by the Research Ethics Committee of the University Center. A total of 200 CBCT exams of individuals of both genders, with at least 18 years of age, were obtained from the image archive of the surgery department of the University Center. An i-CAT Classic was performed using the following parameters: flat panel detector, 0.3 voxel, 0.50 mm focal point, 120 V, 18.45 mAs, 20 s, (Imaging Science International, Hatfield, Pennsylvania, USA). All analysis and measurements were done in an appropriate room through a proper FlexScan S2000 monitor, 20” (Eizo Nanao Corporation, Hakusan, Japan), by i-CAT Vision^®^ Software.

In the parasagittal reformations, the following elements were evaluated: (1) the interforaminal distance, (2) the distance between the apex of the anterior teeth and the beginning of the cortical base of the mandible and (3) the distance between the buccal surface of the cortical bone and the lingual surface of the lingual cortical.

**Figure 1:**
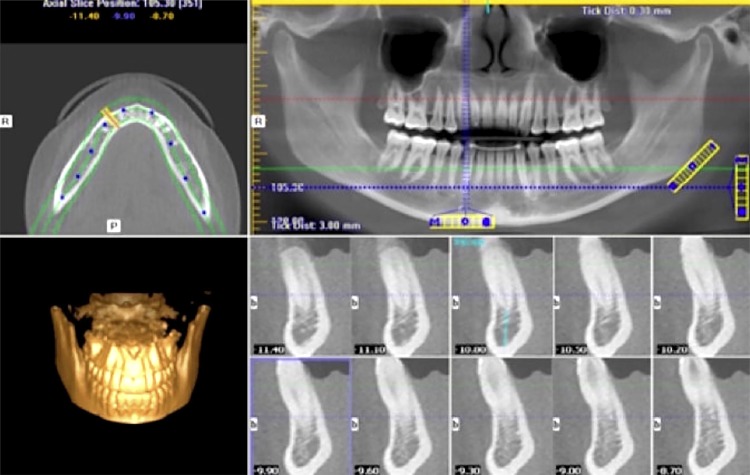
Axial, panoramic and parasagittal reformations and 3D reconstructions on i-CAT Vision® software screen

Panoramic reformation was used to aid in the location of the parasagittal cuts, to visualize the mental foramen, canines and midline and to assess the presence and extension of the anterior loop of the mental foramen ( Figure 1 ).

## Results

Out of 200 patients whose exams were analyzed, 105 were female and 95 were male, their ages varied from 18 to 78 years old and the average age was 43.76 years old.

The anterior loop of the mental nerve was visualized in 47 images (23.5%), bilaterally in 36 images (18.0%), unilaterally on the right side in 3 images (1.5%) and unilaterally on the left side in 8 images (4%). The mean distance measured between the anterior loop of the mental foramen and the base of the mandible was 7.02 mm on the right side and 6.73 mm on the left side, the mean interforaminal distance was 42.27 mm and the mean height was 4.87 mm, as can be seen in Table 1 .

**Table 1:** Mean, minimum and maximum measures of all distance measurements in the parasagittal reformation, 0.30 mm thickness

	Averages	Minimum average	Maximum average
IF	42.27	33.3	55.2
CB-D	13.03	5.92	20.6
ESPM-D	5.82	2.1	8.75
ESPC-D	10.31	4.8	14.7
LM	17.86	9.68	28.28
ESPM-LM	5.93	2.4	9.3
ESPG-LM	10.5	5.6	16.5
CB-E	12.87	5.66	20.7
ESPM-E	5.54	2.4	10.25
ESPC-E	10.07	4.74	14.95
AACB-D	7.02	3	13.8
AACB-E	6.73	3.3	14.7

IF - inter-foramen; BC-R – base-canine - right side; MT-R - medullary thickness - right side; CT-R - cortical thickness – right side; ML - midline-base; MT-ML medullary thickness - midline; CT-ML - cortical thickness - midline; BC-L – base-canine - left side; MT-L - medullary thickness - left side; CT-L - cortical thickness – left side; ALBC-D - anterior loop of the mental foramen base-canine - right side; ALBC-E - anterior loop of the mental foramen base-canine - left side

### External oblique line

#### Samples

A retrospective study was conducted using CBCT exams of patients of both genders, with the minimum age of 18 years old. They were obtained from the same database of i-CAT Classic equipment, flat panel detector, 0.3 voxel, 0.50 mm focal point, 120 v, 18.45 mAs, 20 s, (Imaging Science International, Hatfield, Pennsylvania, USA). The sample size was calculated according to some inclusion criteria. This study was approved by the Research Ethics Committee of the University Center.

#### Measurements

**Figure 2:**
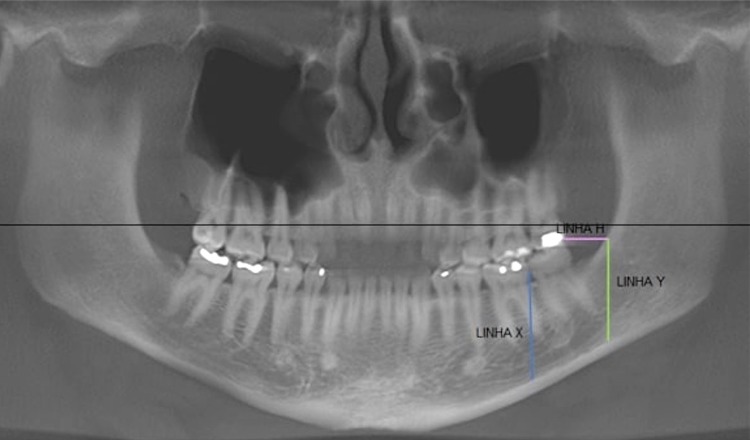
CBCT panoramic reformation showing the positions of Lines X, H and Y, which represent the limits of interest in the evaluation

All analyses and measurements were performed in an appropriate room through a proper monitor FlexScan S2000, 20” (Eizo Nanao Corporation, Hakusan, Japan), by i-CAT Vision^®^ Software. The area submitted to the volumetric calculations was selected based on the region of interest of the graft, being established according to the following limits: Line X = vertical line that tangents the distal of the crown of the first lower molar; Line H = horizontal line that tangents the highest cuspid of molars; Line Y = vertical line that starts where line H crosses the anterior border of the ascending mandibular ramus ( Figure 2 ). The volume was calculated through the expression V = H x L x T where H = height, L = length and T = thickness.

**Figure 3:**
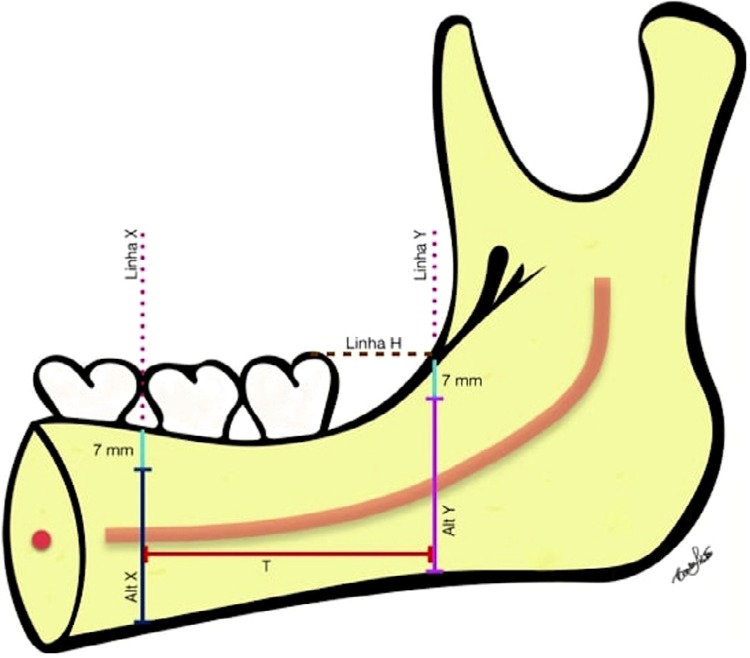
Illustration of the height and length of the graft block

Simulating a donor site for grafting, the height (H) of the bone block was calculated on the panoramic reformation through the distance from the alveolar bone crest to the internal cortical bone of the mandible base in Line X and, in Line Y, subtracting 7 mm to the amount of bone required for the maintenance of the molars and then calculating the average of these measurements. After the measurements, the mean between the heights X and Y was calculated. The length (L) of the bone block was calculated through the distance between Line X and Line Y ( Figure 3 ). The thickness of the hypothetical bone block was calculated in the CBCT parasagittal reformations. The buccal cortical bone thickness was measured both in Line X and in Line Y, in three heights separated by 5 mm, that is: (1) 7 mm, (2) 12 mm and (3) 17 mm below the vestibular alveolar bone crest. After obtaining these values, the average thickness on Line X and on Line Y and the average thickness between X and Y were calculated, resulting in the average thickness of the buccal cortical bone. Through these measurements, the bone volume available on the right side, on the left side and in total were calculated and expressed in cubic millimeters (mm^3^).

**Figure 4:**
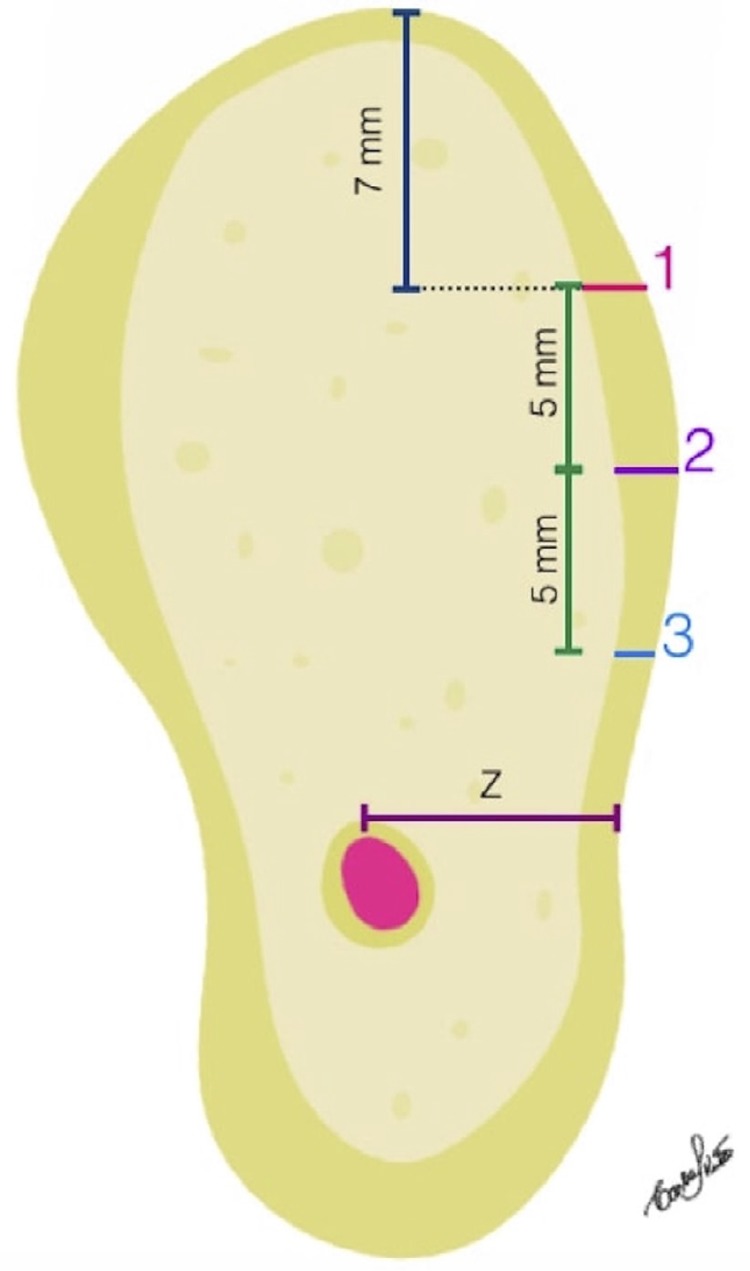
Illustration of a CBCT parasagittal reformation showing the positions of points 1, 2, 3 and of Line Z

Furthermore, the distance from the center of the upper cortical of the mandibular canal (Line Z) to the buccal cortical bone was measured on Line X and on Line Y ( Figure 4 ).

## Results

The samples used in this study were images obtained from 70 patients, with ages between 18 and 68 years old (an age average of 29.61 years old), of which 46 were women and 24 were men. The average values of the measurements were: between the linear distances X and Y: 18.98 mm; height on X and on Y = 17.33 mm and, considering the thickness of the cortical bone, the average of the region (X and Y) was 2.6 mm. The minimum, maximum and average values and the standard deviation are shown in Table 2 . Regarding the volume determination through linear values, the average bone volume available in the posterior region of the mandible was 859.26 mm^3^

**Table 2:** Cortical, medullary and total (cortical + medullary) bone thickness and volumes, considering the mean of the 9 points of the site studied. The table shows the result of the correlation test between the bone thickness and the age of the individuals

	Measurement averages	Standard deviation
Distance of linear lengths X and Y	18.98 mm	18.9±0.12
Heights at X and Y	17.33 mm	17.33±3.00
Cortical bone thickness	2.6 mm	2.60±0.01

Bone volume average 859.26 mm^3^

### Cranial bone

Fifty CBCTs of individuals of both genders with the minimum age of 18 years old were obtained from the image files of the surgery department of the University Center. The sample size was calculated according to some inclusion criteria. This study was approved by the Research Ethics Committee of the University Center. All the exams were performed on an i-CAT Classic (Imaging Science International, Hatfield, Pennsylvania, USA), which has a flat panel detector, with the following acquisition protocol: voxel 0.3 mm, focal point 0.50 mm, 120 V, 18.45 mAs, 20 s. All analyses and measurements were performed in a suitable room on a FlexScan S2000, 20" monitor (Eizo Nanao Corporation, Hakusan, Japan) using the Software i-CAT Vision^®^.

**Figure 5:**
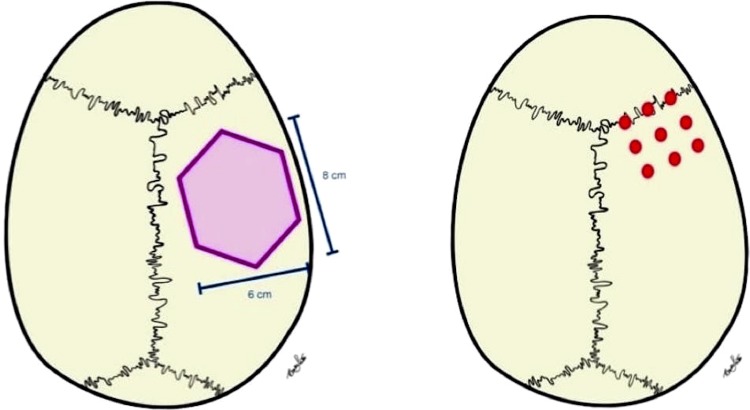
Illustration of the donor site of hexagonal shape described in the study by De Ceulaer, et al.31 (2012). Illustration of the 9 points where the measurements were made

In order to calculate the bone volume that can be obtained, an area of hexagonal shape, 8 cm long and 6 cm wide, similar to that described by De Ceulauer and Abelos^14^ (2012) ( Figure 5 ), was considered as corresponding to the donor site .

Initially, the area of this hexagon was calculated. Next, the cortical, medullary and total bone thickness (cortical + medullary) were measured at 9 points ( Figure 5 ), obtaining the mean bone thickness. By multiplying the area of the hexagon by the bone thickness, the volume of bone that can be removed for grafts from that region was obtained.

**Figure 6:**
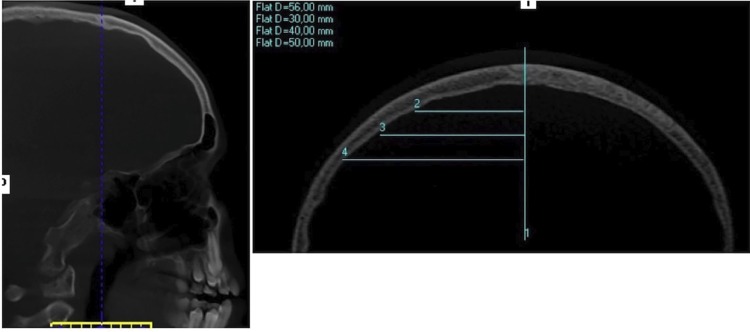
Blue line on the coronal suture. Guide for the measurement of bone thickness, 30, 40 and 50 mm to the right of the sagittal suture

**Figure 7:**
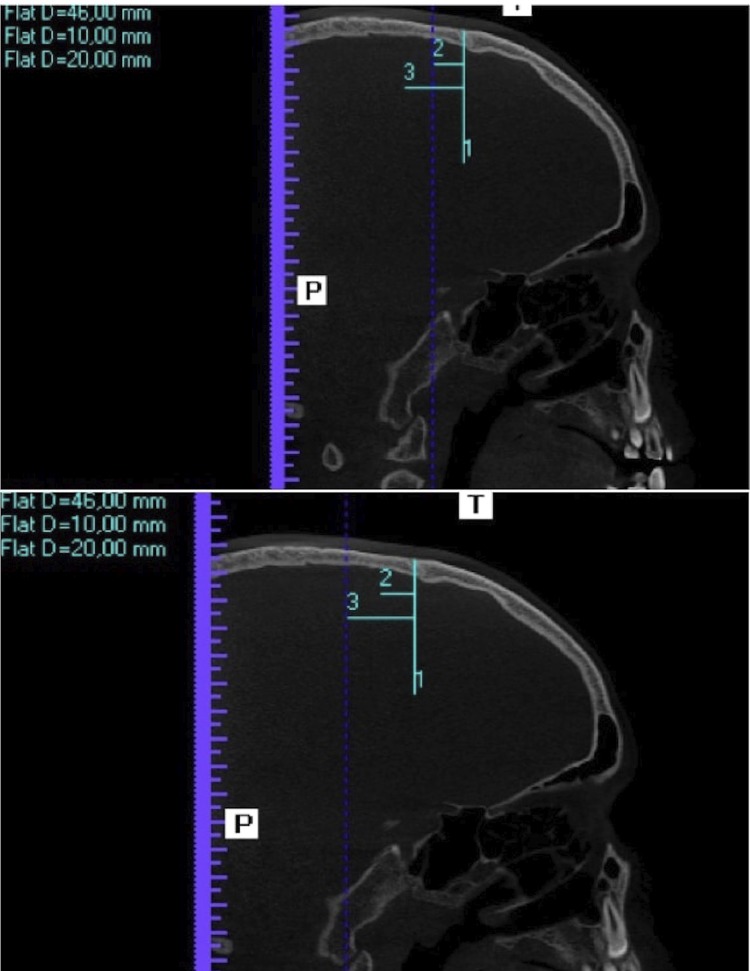
Blue line displaced 10 mm posterior to the coronal suture. Blue line displaced 20 mm posterior to the coronal suture

For the thickness measurements, on the MPR screen of the software, in the window corresponding to the sagittal reformations, the blue line, which determines the coronal reformations, was positioned exactly on the coronal suture ( Figure 6 ). Therefore, a coronal reformation was obtained at the level of the coronal suture, in which a vertical line corresponding to the median sagittal suture was drawn using the distance tool (vertical line) with 30, 40 and 50 mm from the right side, respectively, obtaining a distance guide to the median sagittal suture, to perform the thickness measurements ( Figure 7 ).

In each of these positions, cortical, medullary and total (cortical + medullary) bone thickness measurements were performed at the level of the coronal suture. Afterwards, the blue line, which determines the coronal reformations, was moved first 10 mm, then 20 mm posteriorly and the cortical, medullary and total (cortical + medullary) bone thickness were measured again ( Figure 6 ). In summary, cortical, medullary and total (cortical + medullary) bone thickness were measured at 3 points at the level of the coronal suture, at 3 points 10 mm posteriorly and at 3 points 20 mm posteriorly, as shown in Figure 2 .

## Results

The ages of the 50 patients (25 women and 25 men) whose exams were used in this study ranged from 18 to 71 years old, with an average age of 35.6 years.

Table 3 shows the averages of cortical, medullary and total (cortical + medullary) bone thickness measurements used to calculate the bone volume.

**Table 3:** Comparison of the cortical, medullary and total (cortical + medullary) bone thickness in the 9 points of the area studied, by gender. The table shows the averages of measurements of the cortical, medullary and total (cortical + medullary) bone thickness according to the gender of individuals.

	Mean E	Volume	Standard Deviation
Cortical Bone	2.78 mm	1.167.60	158.76
Medullary Bone	3.00 mm	1.260	443.004
C-M Bone	5.95 mm	2.499	694.992

*Statistically significant

## Discussion

### Mandibular symphysis

The imaging test of the symphysis is necessary to verify if there is enough bone to be used as graft.^13^ With the frequent use of CBCT, which offers more precision and detail, a great variation in the anatomy and dimensions of this region is identified. This proves it to be an important instrument for surgical planning, minimizing intercurrences and complications.^1^

The removal of bone from the mandibular symphysis for grafting is a surgical procedure and the region is completely repaired after 24 months, with the formation of a new cortical and the stabilization of the bone remodeling. It is possible, then, to perform a new intervention in the same region if necessary.^13 , 15^

A safety margin of at least 5.00 mm to the apex of the lower anterior teeth is indicated to avoid sensitivity loss in these teeth.^2^ Experiments with animals have shown that the safety margin should be at least 8.00 mm.^16^ The main advantage of the 8 mm safety margin to the apex of the roots is the 75% reduction of injury possibility in the incisive nerve.^17 , 18^

One recommends to maintain the total integrity of the base of the mandible, preserving the preoperative contour of the chin region and the facial profile, leaving the inferior margin of the symphysis intact and maintaining the midline protrusion, avoiding deformations and irregularities.^16 , 19 , 20^ A 2004 study reported that none of the patients complained about morphology alteration of the chin after the removal of grafts from the mandibular lower anterior region when these recommendations were respected.^2^

This study used as safety margins (1) an 8.00 mm distance from the apexes of the roots of the anterior teeth, (2) the total preservation of the cortical at the base of the mandible, (3) a distance of 5.00 mm anteriorly to the mental foramen and (4) a depth limited to 4.00 mm from the cortical vestibular. The average amount of available bone in the mandibular symphysis region obtained was 628.61 mm.^4^

With the use of the CBCT, considering these safety margins and a correct planning, our study reveals that an adequate patient selection and a reduction in postoperative complications are predictable.^16 - 19 , 21^

The symphysis may provide adequate bone grafts to increase a site previously occupied by two to six teeth. It will never offer enough bone to raise an arch. If the increase in the complete dental arch is required or if the extent of the alveolar bone loss is significant, another source of bone should be considered.^13^

### External oblique line

The use of autogenous bone from the mandibular body and ramus has been proved to be effective in reconstructive surgeries of the maxillary bones.^22^ However, no studies report safe bone volume obtained in this region.^5 , 22^ Furthermore, the posterior region of the mandible, unlike the mandibular symphysis, does not present defined limits for bone removal, so no protocol delimits the exact donor site and there is no standard for the available volume.

In this study, we used the molar teeth as reference for the anterior limit,^23 - 25^ specifically the distal of first molar,^6 , 22^ which is considered a safe limit to prevent interference with the mental nerve ramus. As for the upper limit,^24^ , Capelli^6^ (2003) indicates a distance from 4 to 6 mm medially to the oblique line; and Haggerty, et al.^25^ (2015) says that the superior margin of the graft coincides with the external oblique line. However, in this study, a 7 mm safety margin to the alveolar bone crest was recommended so that the removed bone would not be close to the cervical of the teeth. For the posterior limit, the reference was the exact place where the occlusal plane touches the anterior edge of the ascending mandibular ramus. If the removal of the patch was too high, the osteotomy could injure the buccal artery or expose adipose tissue. Fujita and Shintani^22^ (2015) consider the mandibular lingula as the posterior limit. In the studies by Capelli^6^ (2003), incisions were made at the base of the coronoid process, as well as in the reports by Haggerty et al.^25^ (2015), in which the extension in the posterior direction can also include this region.

For the lower limit, the reference considered is the junction between the anterior and posterior osteotomies, with an average height of 1 cm^6^ or the junction of the osteotomies that extend from 10 to 12 mm below the external oblique line or 4 mm above the mandibular canal.^25^ In this study, the internal cortical of the mandibular base is considered the lower limit. In Line X, or anterior limit, the average height was 16.31 mm and in Line Y, or posterior limit, it was 18.36 mm. The resulting average graft height was 17.33 mm.

The average distance between the anterior and posterior limits (Line X and Line Y) was 18.98 mm, as listed in Table 2 . The average cortical thickness was 2.6 mm, ranging from 1.05 to 4.65 mm. Based on the linear values, the resulting average of bone volume available in the posterior region of the mandible was 859.26 mm^3^ ( Table 2 ).

Some authors^22^ performed a very similar methodology, using the same references of this study (the distal of the first molar, then the distal of the second molar, 10 mm distally to the second molar and 15 mm distally to the second molar). The resulting values of length, height and thickness were respectively: 26 mm, 10 mm and 2 mm. When comparing them to the values of our study, the difference comes from the fact that they evaluate site located a little further in the posterior direction; therefore, these authors present higher length values and lower thickness values.

### Cranial bone

The selection of the graft donor site is based on (1) the amount of bone needed in the recipient bed, (2) the number and location of the implants and (3) the acceptance of the risk of complications by the patient.^25^

Pensler and McCarthy^26^ (1985) studied the thickness of the skullcap in the region of the parietal and occipital bones and found it varied from 6.80 mm to 7.72 mm. In another study carried out in the Anatomy laboratory of the School of Dentistry of the Universidade Estadual Paulista, 49 dry skulls of adult individuals were evaluated.^27^ In that study, all skulls had the cranial vault sectioned at the height of the temporal bone and measured at 4 different points using a goniometer. The average thickness observed was 4.8 mm, 4.5 mm, 6.1 mm, 4.2 mm, respectively, at the 4 evaluated points.^28^ Bernardino Junior, et al.^29^ (2011) measured the thickness of the skullcap at the most protruding point of the parietal tuber. They measured 60 macerated human skulls at the Federal University of Uberlândia, obtaining an average thickness of 5.16 mm.

The most comprehensive study on the subject measured 40 points on 281 dry skulls from the Cleveland Museum of Natural History. The mean thickness found was 6.3 mm, with values ranging from 5.3 mm to 7.5 mm. The site of greatest thickness was the posterior parietal region.^28^

Comparing the results of this study with those found in the literature, a significant difference in the methodology should be considered, since in all the previously mentioned studies performed direct measurements in dry skulls. This means that these thickness measurements considered the external cortical, the medullary bone and the inner cortical bone. In the methodology of this study, on the other hand, only the external cortical and the medullary layer were measured, since they are the ones that are effectively used in the grafts. As it can be seen in Table 3 , the mean thickness of the cortical + medullary bone of the 9 evaluated points was 5.95 mm.

The bone volume of the other donor sites were 628.61 mm^3^in the mandibular symphysis and 859.33 mm^3^ in the external oblique line region. The available bone volume in the skullcap region, calculated in this study was 2,499 mm^3^ ( Table 3 ). Comparing it with the volumes available in the intraoral donor sites of the symphysis and of the posterior region of the mandible, it is reported that the skullcap can offer bone volume almost 3 times greater than the latter and at least 2 times more than the former. In addition, as it allows the withdrawal of several blocks, the skullcap can be used for reconstructions that need more extension.

## Conclusion

All sites discussed in this article are excellent options for the removal of autogenous bone grafts for the reconstruction of defects and for the bone resorption of the jaws. The choice of the site will depend on the type of defect.

Compared with intraoral donor sites, the bone availability of the skullcap is 3 times greater than that of the posterior region of the mandible and at least 2 times greater than that of the mandibular symphysis.
